# Identification of the minimal active soluble TREM2 sequence for modulating microglial phenotypes and amyloid pathology

**DOI:** 10.1186/s12974-021-02340-7

**Published:** 2021-12-10

**Authors:** Xuan Sheng, Yunling Yao, Ruizhi Huang, Ying Xu, Yifei Zhu, Linting Chen, Lianshuai Zhang, Wanbing Wang, Rengong Zhuo, Dan Can, Che-Feng Chang, Yun-wu Zhang, Huaxi Xu, Guojun Bu, Li Zhong, Xiao-Fen Chen

**Affiliations:** 1 Fujian Provincial Key Laboratory of Neurodegenerative Disease and Aging Research, Institute of Neuroscience, School of Medicine, Xiamen University, Xiamen, 361102 China; 2 Xiamen Key Laboratory of Chiral Drugs, School of Medicine, Xiamen University, Xiamen, 361102 China; 3 Shenzhen Research Institute of Xiamen University, Shenzhen, 518063 China; 4 Graduate Institute of Physiology, College of Medicine, National Taiwan University, Taipei, 10051 Taiwan; 5 Department of Neuroscience, Mayo Clinic, Jacksonville, FL 32224 USA

**Keywords:** Microglia, Amyloid plaque, sTREM2

## Abstract

**Background:**

TREM2 is a microglial receptor genetically linked to the risk for Alzheimer’s disease (AD). The cerebrospinal fluid (CSF) levels of soluble TREM2 (sTREM2) have emerged as a valuable biomarker for the disease progression in AD and higher CSF levels of sTREM2 are linked to slower cognitive decline. Increasing sTREM2 in mouse models of amyloidosis reduces amyloid-related pathology through modulating microglial functions, suggesting a beneficial role of sTREM2 in microglia biology and AD pathology.

**Methods:**

In the current study, we performed serial C- and N-terminal truncations of sTREM2 protein to define the minimal sequence requirement for sTREM2 function. We initially assessed the impacts of sTREM2 mutants on microglial functions by measuring cell viability and inflammatory responses. The binding of the sTREM2 mutants to oligomeric Aβ was determined by solid-phase protein binding assay and dot blot assay. We further evaluated the impacts of sTREM2 mutants on amyloid-related pathology by direct stereotaxic injection of sTREM2 proteins into the brain of 5xFAD mice.

**Results:**

We found that both sTREM2 fragments 41–81 and 51–81 enhance cell viability and inflammatory responses in primary microglia. However, the fragment 51–81 exhibited impaired affinity to oligomeric Aβ. When administrated to the 5xFAD mice brain, the sTREM2 fragment 41–81, but not 51–81, increased the number of plaque-associated microglia and reduced the plaque deposition. Interestingly, the fragment 41–81 was more efficient than the physiological form of sTREM2 in ameliorating Aβ-related pathology.

**Conclusions:**

Our results indicate that the interaction of sTREM2 truncated variants with Aβ is essential for enhancing microglial recruitment to the vicinity of an amyloid plaque and reducing the plaque load. Importantly, we identified a 41-amino acid sequence of sTREM2 that is sufficient for modulating microglial functions and more potent than the full-length sTREM2 in reducing the plaque load and the plaque-associated neurotoxicity. Taken together, our data provide more insights into the mechanisms underlying sTREM2 function and the minimal active sTREM2 sequence represents a promising candidate for AD therapy.

## Background

Microglia are the resident immune cells in the central nervous system that are actively involved in the development of neuroinflammatory and neurodegenerative diseases [1]. Large-scale genome-wide association studies have identified multiple polymorphisms on a number of microglia-expressed genes that contribute to late-onset AD risk [2]. Studies on these microglia-specific genetic risk factors have aided the rapid progress towards unraveling the multiple facets of microglia in AD [3]. One such example is provided by the triggering receptor expressed on myeloid cells 2 (TREM2) whose mutations have been identified as major risk factors for late-onset AD [4, 5]. In recent years, the research community has focused its attention on the central roles of TREM2 in microglia biology and AD pathology.

TREM2 is a transmembrane receptor of the immunoglobulin superfamily that is specifically expressed on microglial cell surface. The ectodomain of TREM2 undergoes proteolytic shedding by ADAM10/ADAM17 at the H157-S158 peptide bond resulting in the release of soluble TREM2 (sTREM2) into the extracellular space [6–9]. The levels of sTREM2 in the cerebrospinal fluid (CSF) are increased in AD years before the onset of dementia symptoms, and peak at the mild cognitive impairment (MCI) stage of AD [10]. Higher CSF sTREM2 levels are associated with slower subsequent cognitive and clinical decline in symptomatic AD patients [11, 12]. More recently, the levels of CSF sTREM2 were shown to be negatively correlated with pathological amyloid and tau accumulation [13]. Taken together, these observations suggest a protective role of sTREM2 against AD pathology.

Our previous studies provide evidences supporting the active roles of sTREM2 in regulating microglial dynamics and AD-associated pathology [14, 15]. We found that sTREM2 reduces amyloid plaque load and rescues the functional deficits of spatial memory and long-term potentiation (LTP) of 5xFAD transgenic mice in a microglia-dependent manner. Importantly, sTREM2 enhances an array of microglial functions, including survival, proliferation, migration, clustering in the vicinity of amyloid plaques, and the uptake and degradation of Aβ. To provide more insights into the functional mechanism of sTREM2, we performed serial truncations of sTREM2 sequence and evaluation of sTREM2 variants in regulating microglial activities and amyloid pathology. We defined the minimal sequence requirement for sTREM2 function that spans the N-terminal amino acids H41 to S81 of TREM2. Interestingly, this minimal sTREM2 sequence was more potent than the full-length sTREM2 in reducing plaque load and the plaque-associated neurotoxicity. Our mutagenesis study also underpins the importance of sTREM2 interaction with Aβ that enhances the microglial clustering to the vicinity of an amyloid plaque and reduces the plaque load. Taken together, our data provide more insights into the mechanisms underlying sTREM2 function and the minimal active sTREM2 sequence represents a promising candidate for AD therapy.

## Methods

### Mice

The 5xFAD mice were purchased from the Jackson Laboratory. Both male and female mice were used in this study. Mice were randomly selected for further biological analysis, and the researchers were blinded to group allocation during the experiments or result assessments.

### Stereotaxic injection and preparation of brain samples

Mice were anesthetized with isoflurane in a cage and placed in a stereotaxic frame (RWD Life Science) as described in our previous study [16]. The anesthesia was maintained using isoflurane (1–2%) inhalation, then a skin incision was made, and holes were drilled at x (± 2.0 mm from bregma) and y (− 2.0 mm from bregma). We used a 5 μL Hamilton syringe (7634–01) with a 30G gauge needle (7803–07) fixed on a stereotaxic frame and connected to a microinjection pump (RWD Life Science). The Fc-labeled sTREM2 fragment or Fc was injected into the right and left hippocampi of 5xFAD (7-month-old) mice at 0.20 µL/min and 2.0 mm z-depths, respectively. The syringe was left for 5 min after each injection and then being slowly withdrawn. Seven days after the injection, the mice were anesthetized and perfused with ice-cold PBS. For immunohistochemistry analysis, brains were dissected out quickly and fixed in 4% paraformaldehyde (PFA) at 4 °C overnight, transferred to 30% sucrose for 48 h, embedded and frozen in Tissue-Tek® O.C.T. Compound (SAKURA, 4583) at − 80 °C.

### Primary microglial cultures

Primary microglial cultures were prepared as described in our previous study [15]. Briefly, mice at postnatal day 1–2 were used to prepare mixed glial cultures. Cells were plated onto poly-L-lysine-coated flasks and grown in DMEM supplemented 10% heat-inactivated fetal bovine serum (FBS) and 1% penicillin streptomycin solution (Gibco, 15140–122). After 3 days, medium was changed to DMEM that contained 25 ng/mL GM-CSF (R&D Systems, 415-ML-050) and 10% FBS. Primary microglia were harvested by shaking (200 rpm, 20 min) after 10–12 days in culture and once every 3 days thereafter. Primary microglia were cultured in DMEM supplemented with 10% FBS before any treatments.

### Real-time quantitative PCR analysis

Total RNA was extracted from primary microglia treated with the sTREM2 fragments using TRIzol reagent (Invitrogen). One microgram total RNA was reverse-transcribed into cDNA using ReverTra Ace qPCR RT Master Mix (TOYOBO). Quantitative PCR was performed using SYBR Green Master mix (Roche). The primer sequences for β-Actin, IL-1β and TNF were as follows: β-Actin forward, 5′-AGTGTGACGTTGACATCCGTA-3′, β-Actin reverse, 5′-GCCAGAGCAGTAATCTCCTTC-3′, IL-1β forward, 5′-GCAACTGTTCCTGAACTCAACT-3′, IL-1β reverse, 5′-ATCTTTTGGGGTCCGTCAACT; TNF forward, 5′-GTCTACTGAACTTCGGGG-3′, TNF reverse, 5′-CTGAGTGTGAGGGTCTGGGC-3′. All primer sets were purchased from Life Technologies.

### TUNEL assay

Primary microglia were seeded on poly-lysine-coated glass coverslips with a density of 1 × 10^5^ cells/well in 24-well culture plates and cultured for 48 h after GM-CSF withdrawal. Microglia were incubated with each sTREM2 fragment at 20 nM for 24 h in DMEM without serum and penicillin streptomycin. After washing three times with 1 × PBS, cells were fixed with 4% PFA for 15 min and then permeabilized with 0.2% Triton X-100 for 5 min. Terminal deoxynucleotidyl transferase-mediated dUTP nick end-labeling (TUNEL) assay was performed according to the manufacturer’s protocol (Promega). Cells were stained with DAPI (1 μg/mL) for 10 min and washed with 0.1% PBST for 15 min. Coverslips were mounted on glass slides and observed under microscope (Olympus FV1000MPE-B).

### Dot blot

The fragments of sTREM2 diluted in PBS were spotted onto a methyl alcohol-treated PVDF membrane using a dot blot manifold apparatus (GE Healthcare). Membrane was blocked in 1% Block Ace (Bio-Rad) for 2 h, and incubated with 100 nM oAβ42 in PBS overnight at 4 °C. Membrane was blotted by Aβ primary antibody (MOAB-2, Abcam) and detected by a secondary antibody conjugated with horseradish peroxidase (HRP). Bound protein was visualized using ECL Western blotting detection reagents (Millipore).

### Solid-phase binding

The purified proteins of Fc and Fc-tagged sTREM2 fragments with indicated concentrations in PBS were coated to each well of the 96-well plates overnight at 4 °C. Wells were washed 3 times with 0.05% PBST and blocked with 1% Block Ace (Bio-Rad) for 2 h. Two-hundred nM of oAβ42 diluted in PBS were added and incubated at 37 °C for 25 min. After washing with 0.1% PBST, the bound Aβ were detected by Aβ primary antibody and a secondary antibody conjugated with HRP. Finally, plates were washed and developed with 100 μL TMB substrate (Sigma Aldrich), and read at 620 nm.

### Immunohistochemistry

Mouse coronal brain sections with 15 μm thickness were mounted on glass slides, air-dried overnight at 37 °C. The sections were stored at − 80 °C or subsequently processed for staining. The sections were washed 3 times with 1 × PBS and incubated with blocking buffer (5% normal donkey serum and 0.2% Triton X-100 in PBS) for 1 h at room temperature (RT). The sections were then incubated with primary antibodies for 24 h at 4 °C and the Alexa-fluorophore-conjugated secondary antibodies (Thermo Fisher Scientific) for 2 h at RT. Sections were stained with DAPI (1 μg/mL) for 10 min and sealed with an anti-fade reagent (Thermo Fisher Scientific). The following primary antibodies were used: anti-Iba1 (Wako), anti-Aβ (MOAB-2, Abcam) and anti-Lamp1 (DB Biosciences). For Thio-S staining, the sections were incubated with 1 mM Thioflavin-S solution at RT for 8 min.

### Microscopy and analysis

For confocal microscopy, images were scanned and acquired with a NIKON A1R Plus confocal microscope. Imaging parameters were kept constant in the same experiment. For plaque load and microglial area, 7 μm z-stacks consisting of 8 optical slices of 1 μm thickness were captured on a 20 × objective. Images were analyzed using the ImageJ (NIH) by adjusting threshold parameter to highlight all of the interested structures. The parameters were kept consistent throughout analysis. For quantifying the number of plaque-associated microglia, 9 μm z-stacks consisting of 10 optical slices of 1 μm thickness were captured on a 60 × objective. The number of microglia within a 25 µm radius of plaque was determined in a double-blinded manner.

### Statistical analysis

All histological data analyses were performed blindly. Statistical tests were performed using GraphPad software. The statistical methods for each quantitated data were described in the figure legends. All data are shown as mean ± SD and n values were indicated in each figure legend. The significant levels are as follows: ns, not significant; *, *p* < 0.05; **, *p* < 0.01; ***, *p* < 0.001; ****, *p* < 0.0001. *p* < 0.05 was considered as statistically significant.

## Results

### The N-terminal amino acids 1–81 of sTREM2 are sufficient to modulate microglial responses

**Fig. 1:**
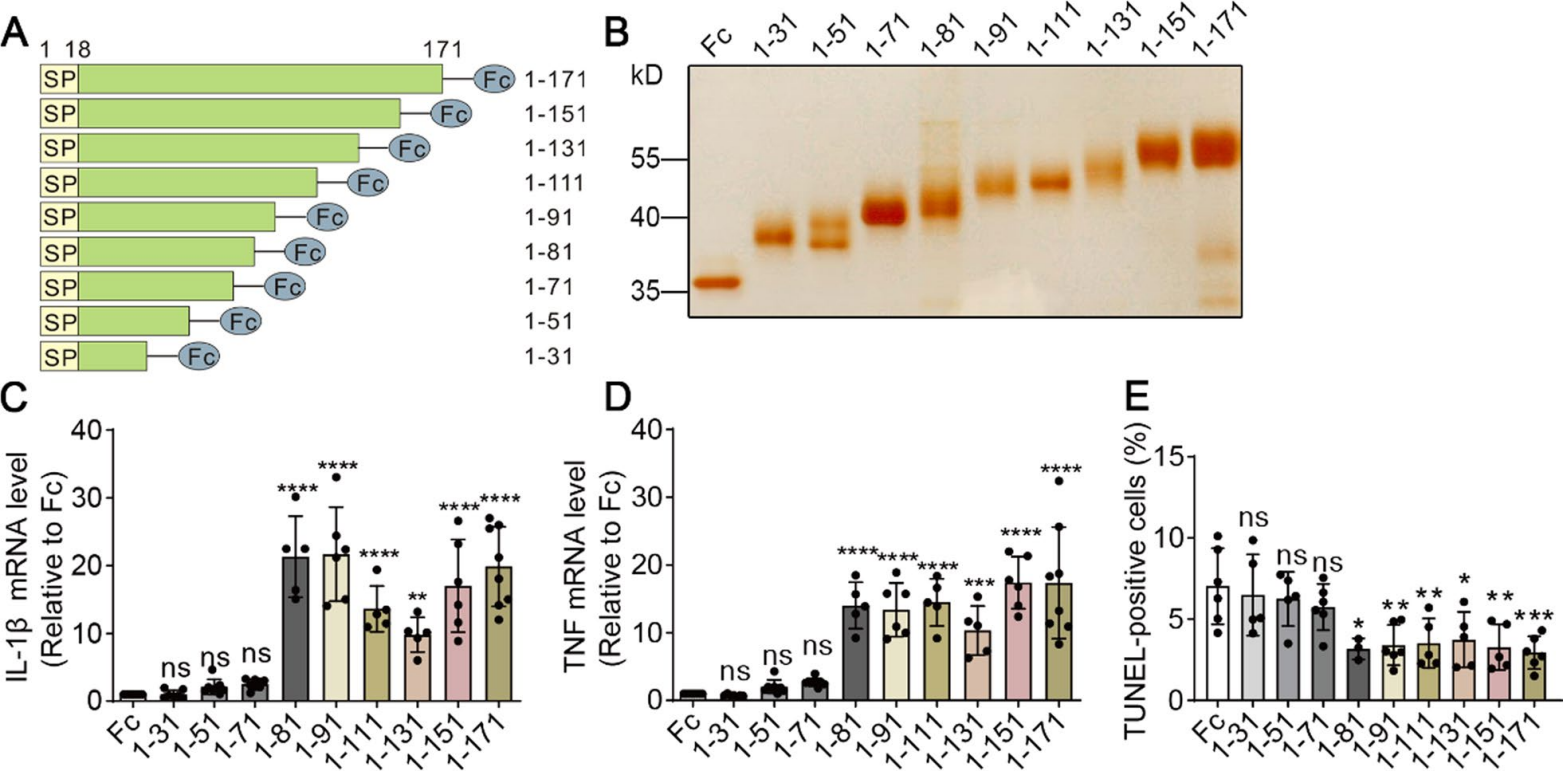
The effects of the C-terminal truncation mutants of sTREM2 on microglial responses. **A** Schematic diagram of a series of C-terminal truncation mutants of sTREM2 fused with human IgG-Fc. **B** Silver staining analysis of the sTREM2 fragments purified from the conditioned media of transfected HEK293T cells. **C**, **D** Primary microglia were treated with 20 nM of Fc alone or the Fc-tagged sTREM2 fragments for 4 h. RNA were extracted, and the relative mRNA levels of IL-1β (**C**) and TNF (**D**) were determined by quantitative real-time PCR. β-Actin was used as an internal control (*n* = 5–8 per group, one-way ANOVA). **E** Microglia were treated with 20 nM of Fc alone or the Fc-tagged sTREM2 fragments for 24 h after GM-CSF withdrawal. TUNEL was performed to analyze cellular apoptosis (*n* = 3–6, one-way ANOVA)

We have previously reported that the recombinant sTREM2 protein (spanning the N-terminal amino acids 1–171) ameliorates amyloid pathology and related toxicity through modulating microglial functions [15, 16]. We performed serial truncations on human sTREM2 (1–171) to define the minimal sequence requirements for sTREM2 function. A series of C-terminal truncation mutants of sTREM2 were fused with human IgG-Fc and purified from the conditioned media of transfected HEK293T cells (Fig. 1A, B). Since our previous study has shown that sTREM2 induces inflammatory responses and enhances cell survival [15], we assessed the impacts of recombinant sTREM2 mutants on these two activities in primary microglia. No significant differences in the levels of IL-1β and TNF were observed between Fc control and the fragments spanning N-terminal amino acids 1–31, 1–51 or 1–71 (Fig. 1C, D). In contrast, the fragments 1–81, 1–91, 1–111, 1–131 and 1–151 significantly increased the expression levels of proinflammatory cytokines similar to the sTREM2 1–171 protein. Next, we assayed the impact of sTREM2 truncation mutants on microglial cell viability. The cellular apoptosis induced by GM-CSF withdrawal was markedly prohibited by the presence of sTREM2 mutants 1–81, 1–91, 1–111, 1–131 and 1–151 (Fig. 1E). However, this protective function was not observed with the administration of sTREM2 mutants 1–31, 1–51 and 1–71. Collectively, our data suggest that the N-terminal amino acids 1–81 of sTREM2 are sufficient to increase the inflammatory responses and cellular survival in microglia.

### The sTREM2 fragment 41–81 is sufficient to modulate microglial responses

**Fig. 2:**
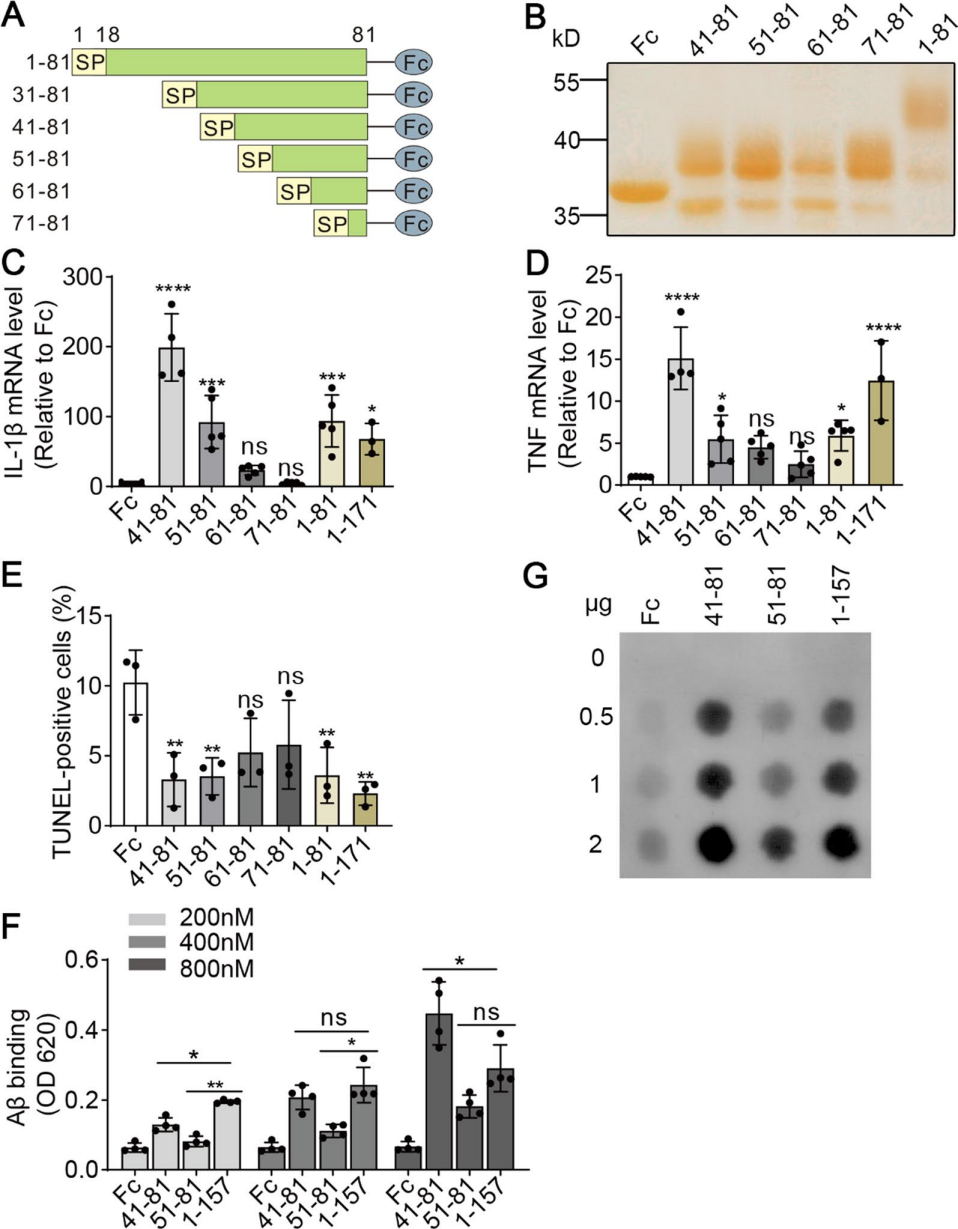
The effects of the N-terminal truncation mutants of sTREM2 on microglial responses. **A** Schematic diagram of a series of N-terminal truncation mutants of sTREM2 fused with human IgG-Fc. **B** Silver staining analysis of the sTREM2 fragments purified from the conditioned media of transfected HEK293T cells. **C**, **D** Primary microglia were treated with 20 nM of Fc alone or the Fc-tagged sTREM2 fragments for 4 h. RNA was extracted, and the relative mRNA levels of IL-1β (**C**) and TNF (**D**) were determined by quantitative real-time PCR. β-Actin was used as an internal control (*n* = 3–5 per group, one-way ANOVA). **E** Microglia were treated with 20 nM Fc alone or the Fc-tagged sTREM2 fragments for 24 h after GM-CSF withdrawal. TUNEL was performed to analyze cellular apoptosis (*n* = 3, one-way ANOVA). **F** The binding affinity between oAβ_1–42_ and either of Fc, the Fc-tagged sTREM2 fragment 41–81, 51–81, 1–157 at indicated concentrations were analyzed by solid-phase binding assay (*n* = 4, one-way ANOVA). **G** Dot blot of oAβ_1–42_ binding to either of Fc, sTREM2 fragment 41–81, 51–81, 1–157. A PVDF membrane was spotted with each of the sTREM2 fragment and then incubated with 100 nM oAβ_1–42_. Bound Aβ was detected by MOAB-2 antibody

The sTREM2 1–81 represents the shortest sequence requirement for sTREM2 function as suggested by our C-terminal truncation study. Next, we performed a series of N-terminal truncations on sTREM2 1–81 to identify the minimal active sTREM2 fragments. A total of five truncation mutants were expressed in the HEK293T cells (Fig. 2A, B). However, the secretion of sTREM2 mutant carrying residues 31–81 into the extracellular space was almost completely abolished, therefore preventing the follow-up protein purification for further analysis. The sTREM2 fragments 41–81 and 51–81 triggered a proinflammatory response when administrated to the primary microglial culture (Fig. 2C, D). In contrast, sTREM2 61–81 and 71–81 failed to stimulate the expression of IL-1β and TNF, indicating their loss of function in mediating microglial activation. Interestingly, similar effects were observed in the cellular apoptosis assay. Both sTREM2 fragments 41–81 and 51–81, but not sTREM2 61–81 and 71–81, significantly suppressed microglial apoptosis in the absence of GM-CSF (Fig. 2E).

The extracellular domain of TREM2 has been reported to bind to the major pathological component of AD, oligomeric amyloid-β 1–42 (oAβ_1–42_) with high affinity [17, 18]. Such an interaction likely enhances the clustering of microglia to the vicinity of the plaque, potentially leading to barrier formation to reduce plaque toxicity and the phagocytic clearance of plaques [16]. A recent study has reported that sTREM2 binds to Aβ oligomers and inhibits Aβ oligomerization and Aβ-induced neurotoxicity, suggesting additional mechanisms by which sTREM2 protects against AD pathology [19]. We further determine the interaction between oAβ_1–42_ and the sTREM2 fragment 41–81 or 51–81. In the solid-phase protein binding assay, we found that oAβ_1-42_ binds to the sTREM2 41–81 and the full-length sTREM2 with higher affinity than the sTREM2 51–81 (Fig. 2F). Similar observations were made in a dot blot assay (Fig. 2G), suggesting an impaired binding of the sTREM2 fragment 51–81 to oAβ_1–42_. Taken together, our serial truncation studies identified a minimal sTREM2 fragment spanning residues 41–81 that recapitulates much of the function of full-length sTREM2 in modulating microglial responses, including inflammation, survival and the interaction with toxic Aβ oligomers.

### The sTREM2 fragment 41–81 reduces the plaque deposition in 5xFAD mouse model of amyloidosis

**Fig. 3:**
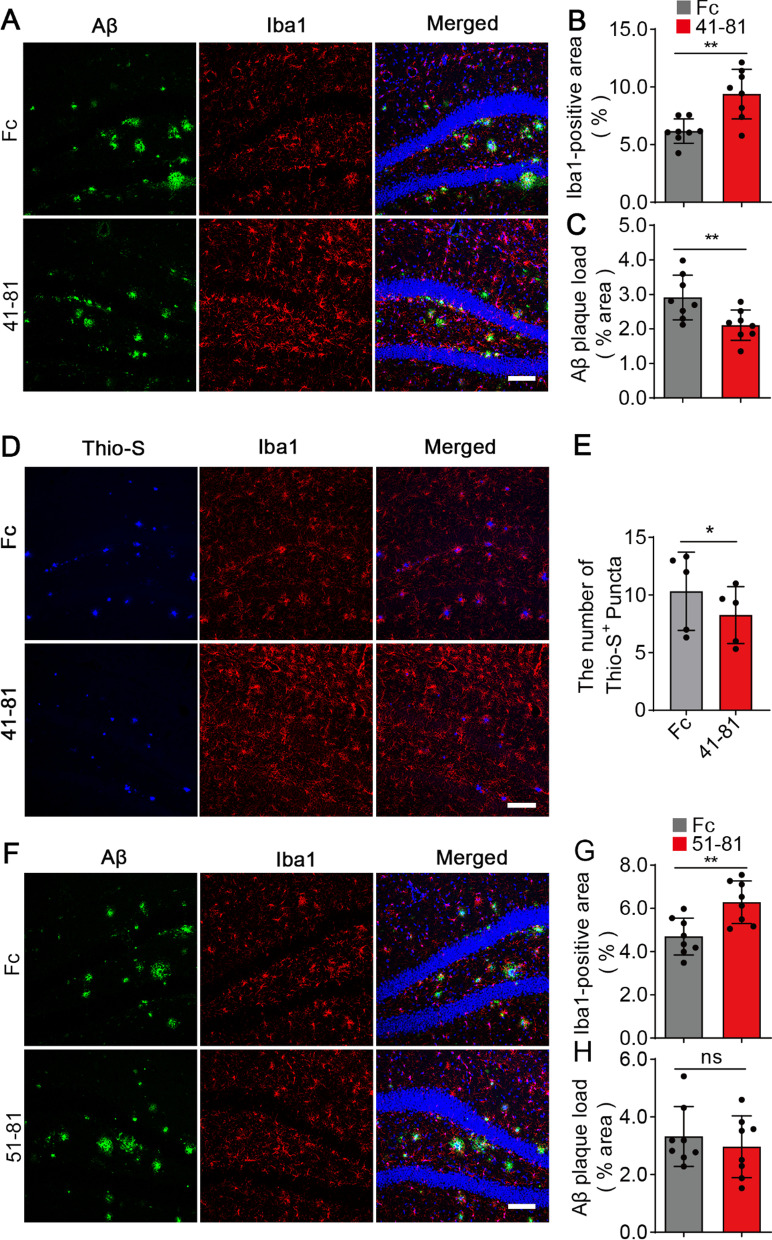
The impacts of sTREM2 fragments on amyloid deposition in 5xFAD mice. **A** and** D** The 7-month-old 5xFAD mice were injected with the Fc-tagged sTREM2 fragment 41–81 or Fc alone into the right and left hippocampi, respectively. **A** Coronal sections were stained with DAPI (blue) for nuclei, MOAB-2 (green) for Aβ, and Iba1 (red) for microglia. **D** Coronal sections were stained with Thio-S (blue) for dense-core plaque, and Iba1 (red) for microglia. Representative z-stack images of the hippocampus regions are shown. Original magnification× 20; scale bar, 100 μm. **B** Quantitation of the Iba1-positive area in A (*n* = 8 mice, 24 fields of each group for analysis, paired Student’s *t* test). **C** Quantitation of the amyloid plaque deposition in A (*n* = 8 mice, 24 fields of each group for analysis, paired Student’s *t* test). **E** Quantitation of the Thio-S-positive area in D (*n* = 5 mice, 15 fields of each group for analysis, paired Student’s *t* test). **F** The 7-month-old 5xFAD mice were injected with the Fc-tagged sTREM2 fragment 51–81 or Fc alone into the right and left hippocampi, respectively. Coronal sections were stained with DAPI (blue) for nuclei, MOAB-2 (green) for Aβ, and Iba1 (red) for microglia. Representative z-stack images of the hippocampus regions are shown. Original magnification× 20; scale bar, 100 μm. **G** Quantitation of the Iba1-positive area in F (*n* = 8 mice, 24 fields of each group for analysis, paired Student’s *t* test). **H** Quantitation of the amyloid plaque deposition in F (*n* = 8 mice, 24 fields of each group for analysis, paired Student’s *t* test)

It has been demonstrated that the full-length sTREM2 protein reduces the amyloid plaque load in 5xFAD transgenic mice in a microglia-dependent manner [16]. Given that the sTREM2 fragment 41–81 recapitulates the in vitro activities of the full-length sTREM2, we further evaluated its impacts on amyloid-related pathology. The sTREM2 fragment 51–81 which promotes microglial survival and inflammatory responses, but has impaired binding to the oAβ was also assessed. We injected the Fc-tagged sTREM2 mutants into the right hippocampus of 7-month-old 5xFAD mice, with Fc alone injected into the left hippocampus as a control. Similar to the full-length sTREM2 protein, the sTREM2 fragment 41–81 markedly increased the Iba1-positive area and dramatically reduced the amyloid plaque load in the ipsilateral hippocampus 7 days after delivery (Fig. 3A–C). The sTREM2 fragment 41–81 treatment also significantly reduced the number of dense-core plaque stained by Thioflavin-S (Fig. 3D, E). Interestingly, the sTREM2 fragment 51–81 significantly increased the area of Iba1 staining 7 days after its delivery to the hippocampus of 5xFAD mice; however, it failed to reduce the amyloid plaque load (Fig. 3F–H).

**Fig. 4:**
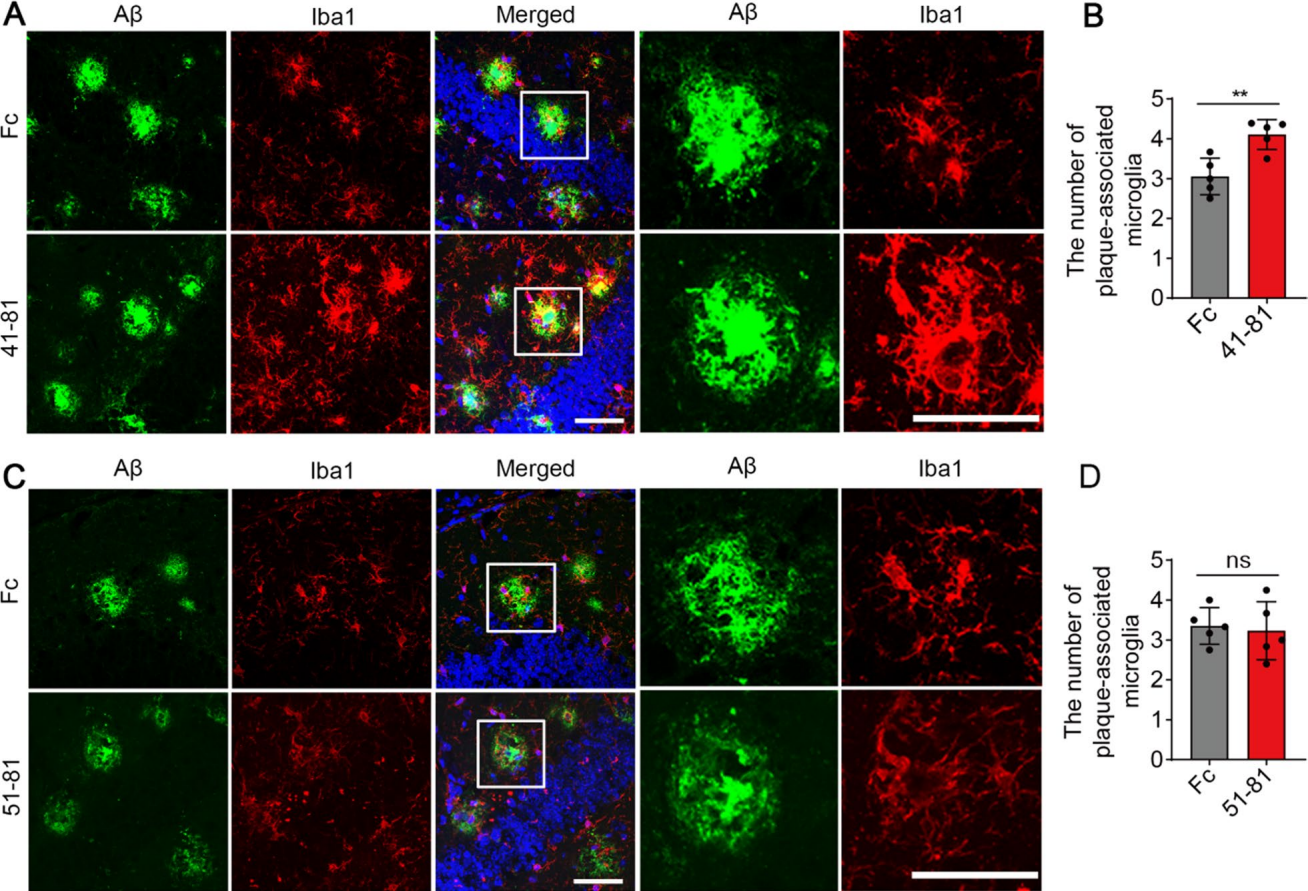
The sTREM2 fragment 41–81 increases the number of plaque-associated microglia. **A**, **C** Coronal sections from the 5xFAD mice injected with the Fc-tagged sTREM2 fragments (**A**, the sTREM2 fragment 41–81; **C**, the sTREM2 fragment 51–81) or Fc alone were stained with DAPI (blue) for nuclei, MOAB-2 (green) for Aβ, and Iba1 (red) for microglia. Representative z-stack images of the hippocampus regions are shown. Original magnification× 60; scale bar, 50 μm. **B** Quantitation of the number of plaque-associated microglia in the presence of sTREM2 fragment 41–81 (*n* = 5 mice, 55 plaques of Fc and 43 plaques of 41–81, paired Student’s *t* test). **D** Quantitation of the number of plaque-associated microglia in the presence of sTREM2 fragment 51–81 (*n* = 5 mice, 47 plaques of Fc and 47 plaques of 51–81, paired Student’s *t* test)

One important feature of the full-length sTREM2 protein is that it enhances the clustering of microglia to the vicinity of the plaque [16]. We therefore quantified the number of plaque-associated microglia upon administration with the sTREM2 fragments. Consistent with its protective effects on plaque deposition, the sTREM2 fragment 41–81 significantly increased the number of microglia in the vicinity of an amyloid plaque (Fig. 4A, B). However, similar effects were not observed with the administration of the sTREM2 fragment 51–81 which has impaired binding to the oAβ_1–42_ (Fig. 4C, D). Taken together, we conclude that the fragment encompassing residues 41–81 of sTREM2 is the minimal sequence requirement for sTREM2 to protect against amyloid pathology. Our findings also indicate that the interaction of sTREM2 truncated variants with oAβ_1–42_ was essential for microglial clustering around amyloid plaque and reducing amyloid pathology.

### The sTREM2 fragment 41–81 is more potent than the full-length sTREM2 in reducing the amyloid-related pathology

**Fig. 5:**
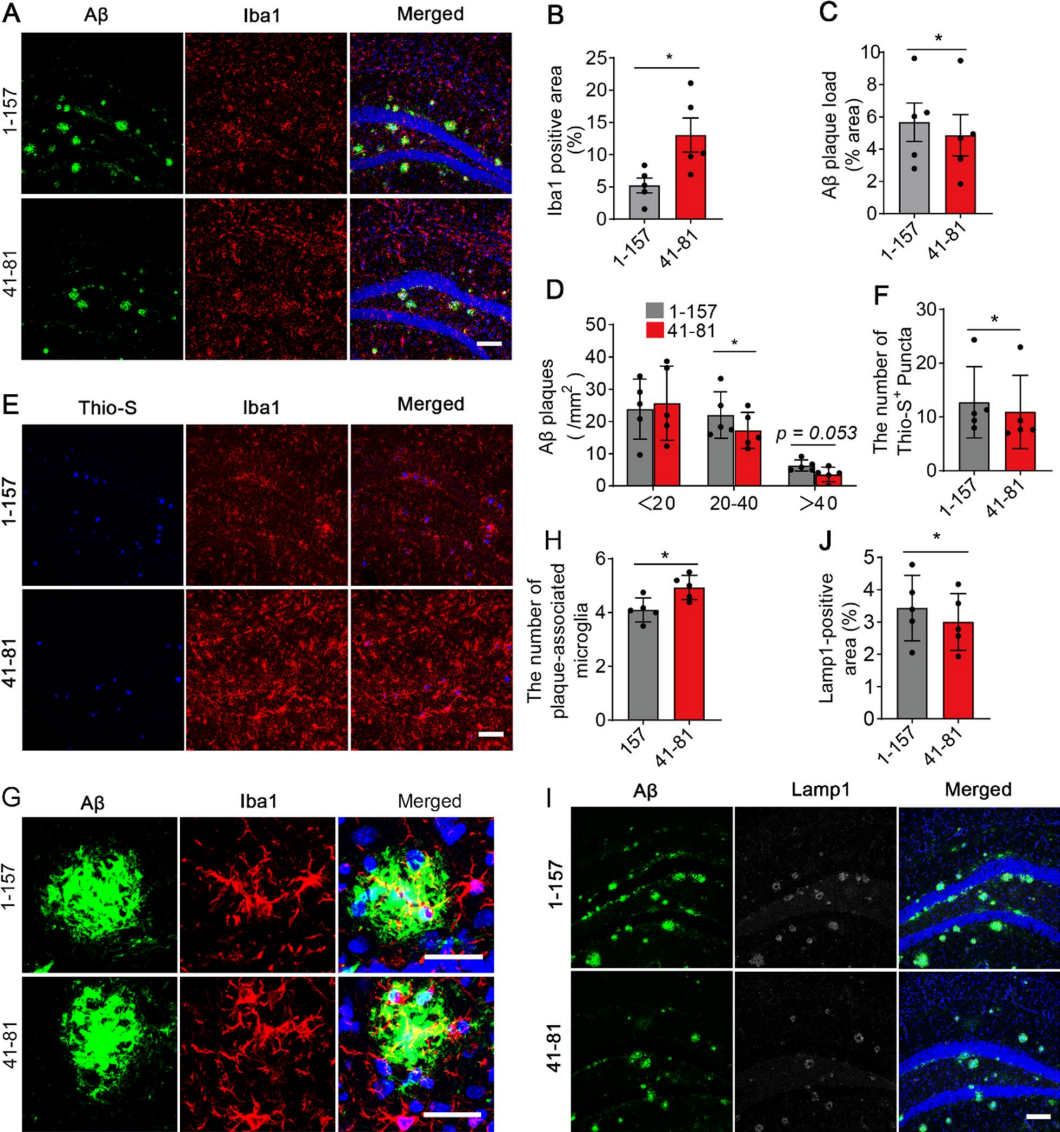
Comparing the impacts of sTREM2 fragment 41–81 and the full-length protein on amyloid plaque deposition in 5xFAD mice. **A** and **E** The 8-month-old 5xFAD mice were injected with the Fc-tagged sTREM2 1–157 or fragment 41–81 into the right and left hippocampi, respectively. **A**, coronal sections were stained with DAPI (blue) for nuclei, MOAB-2 (green) for Aβ, and Iba1 (red) for microglia. **E**, coronal sections were stained with Thio-S (blue) for dense-core plaque, and Iba1 (red) for microglia. Representative z-stack images of the hippocampus regions are shown. Original magnification × 20; scale bar, 100 μm. **B** Quantitation of the Iba1-positive area in A (*n* = 5 mice, 15 fields of each group for analysis, paired Student’s *t* test). **C** Quantitation of amyloid plaque deposition in A (*n* = 5 mice, 15 fields of each group for analysis, paired Student’s *t* test). **D** Quantitation of the number of plaques with different sizes in A (*n* = 5 mice, 15 fields of each group for analysis, paired Student’s *t* test). **F** Quantitation of the Thio-S-positive area in E (*n* = 5 mice, 15 fields of each group for analysis, paired Student’s *t* test). **G** Coronal sections from the 5xFAD mice injected with the Fc-tagged sTREM2 1–157 and sTREM2 fragment 41–81 were stained with MOAB-2 (green) for Aβ, and Iba1 (red) for microglia. Representative z-stack images of the hippocampus regions are shown. Original magnification × 60; scale bar, 50 μm. **H.** Quantitation of the number of plaque-associated microglia in the presence of sTREM2 1–157 or 41–81 (*n* = 5 mice, 54 plaques of 41–81 and 47 plaques of 1–157, paired Student’s *t* test). **I.** Coronal sections from the sTREM2-injected 5xFAD mice were stained with DAPI (blue) for nuclei, MOAB-2 (green) for Aβ and Lamp1 (white) for dystrophic neurites. Representative z-stack images of the hippocampus regions are shown. Original magnification × 20; scale bar, 100 μm. **J** Quantitation of the total area of Lamp1-positive dystrophic neurites in I (*n* = 5 mice, 15 fields of each group for analysis, paired Student’s *t* test)

It was later reported that the generation of sTREM2 is facilitated by the regulated shedding of membrane-bound TREM2 at the H157-S158 peptide bond [7–9]. Hence, the physiological form of sTREM2 protein encompasses the residues 1–157 of TREM2 extracellular domain, which was termed as sTREM2 1–157. To directly compare the activity of the physiological form of sTREM2 and the sTREM2 fragment 41–81 in reducing plaque deposition, we injected the Fc-tagged of either sTREM2 1–157 or the fragment 41–81 into the right and left hippocampi of 5xFAD mice, respectively. Consistent with our in vitro analysis that the sTREM2 fragment 41–81 induces greater magnitude of inflammatory response than the full-length sTREM2 protein, the area of Iba1 staining was significantly increased in the ipsilateral hippocampus injected with the sTREM2 fragment 41–81 (Fig. 5A, B). Moreover, the sTREM2 fragment 41–81 was more efficient than the sTREM2 1–157 in reducing the overall Aβ plaque deposition (Fig. 5A, C). Of note, the number of plaques with 20–40 μm in diameter was markedly reduced by the sTREM2 fragment 41–81, while the number of large plaques (> 40 μm in diameter) exhibited a trend towards a reduction as compared to the sTREM2 1–157 (Fig. 5D). The number of Thioflavin S-positive plaques was significantly lower in the hippocampus administrated with the sTREM2 fragment 41–81, as compared to the sTREM2 1–157 (Fig. 5E, F). Upon quantification of the number of microglia in the vicinity of an amyloid plaque, we found that the sTREM2 fragment 41–81 promotes more microglial clustering than the sTREM2 1–157 (Fig. 5G, H). And the total area of dystrophic neurites as detected by immunostaining with an antibody against Lamp1 was significantly lower in the hippocampus injected with the sTREM2 fragment 41–81 (Fig. 5I, J). We therefore conclude that the sTREM2 fragment 41–81 which we defined through serial truncations is more potent than the physiological form of sTREM2 protein in modulating microglial responsiveness and decreasing the plaque deposition in 5xFAD mice.

## Discussion

In the current study, we conducted serial C- and N-terminal truncations of sTREM2 and defined a 41-amino acid sequence of sTREM2 that is sufficient for modulating microglial functions and ameliorating the amyloid pathology. Importantly, this minimal sTREM2 sequence was more potent than the full-length sTREM2 in lessening the plaque load and the plaque-associated neurotoxicity. Our study also underpins the importance of sTREM2 interaction with Aβ that enhances the microglial clustering to the vicinity of an amyloid plaque and reduces the plaque load.

It has been shown that the binding of TREM2/sTREM2 to Aβ counteracts key in vivo and in vitro pathological effects associated with Aβ. Several studies have demonstrated that the binding of oAβ to TREM2 promotes microglial clustering around oAβ-bearing brain regions, potentially contributing to the barrier function of microglia to compact the plaques and the phagocytic clearance of Aβ plaques [17, 18, 20]. Interestingly, recent studies also reveal that sTREM2 directly binds to the monomeric or oligomeric form of Aβ and blocks Aβ aggregation and neurotoxicity [19, 21]. These findings suggest a protective function for TREM2/sTREM2 interaction with Aβ in the context of amyloid pathology and encourage further research to map the key required residues on TREM2 that mediates its interaction with Aβ.

We have shown previously that the interaction between TREM2 and oAβ_1–42_ relies on the positively charged amino acids within residues 31–91 of TREM2 [18]. In the current study, we further defined the sTREM2 fragment 41–81 that is sufficient for TREM2 binding to oAβ_1–42_. In contrast, the sTREM2 fragment 51–81 exhibits impaired affinity to Aβ oligomers, implicating that the amino acids spanning 41–50 of TREM2 is required for the interaction between TREM2 and oAβ_1–42_. The 41–50 residues cluster into the complementarity-determining region 1 (CDR1) of TREM2 immunoglobulin domain which has been shown to be involved in ligand binding [21, 22]. Interestingly, there is a significant enrichment of positively charged amino acids (arginine and lysine) within residues 41–50 (K42, R46, R47, R48), and their individual mutation into alanine significantly reduces the binding affinity of TREM2 to oAβ_1–42_ [18]. Among these positively charged amino acids, mutation in the R47 constitutes one of the strongest single allele genetic risk factors for AD [23]. Several studies have demonstrated that the TREM2 R47H exhibits reduced binding affinity for Aβ in vitro [17–19] and impairs the interaction of sTREM2 with plaques in vivo [24]. Although these findings support the notion that the key residues within 41–50 of TREM2 are essential for its binding to Aβ, further structural studies are required to elucidate the molecular mechanism underlying such an interaction.

Our studies on a series of truncated mutants of sTREM2 provide new insights into the functional mechanism mediating the ability of sTREM2 to attenuate amyloid pathology. Microglial activation appears to play a protective role in the early stage of amyloid-associated pathology, as supported by observational studies in human AD brain [11, 25–27]. The sTREM2 51–81 mutant successfully induced microglial activation but failed to reduce the plaque load, indicating that other functional aspects of sTREM2 are important for reducing the plaque deposition. Interestingly, the sTREM2 mutant 51–81 which has reduced affinity to oAβ failed to promote microglial clustering around amyloid plaque. Hence, the data support a key role of sTREM2 interaction with oAβ that enhances the recruitment of microglia around amyloid in vivo. Since the microglial clustering to the vicinity of amyloid plaques is a prerequisite for their barrier function and the subsequent uptake and degradation of plaque, the sTREM2 mutant 51–81 may therefore lose its capacity in reducing the amyloid plaque load.

It has been reported that the Aβ oligomers induce TREM2 proteolysis and sTREM2 release [19]. Interestingly, the sTREM2 protein has been shown to colocalize with amyloid plaques in the 5xFAD mice brains [24]. Based on our previous studies and current observations, we propose that sTREM2 released from the cell membranes latches onto amyloid plaque via binding to oAβ, which then induces the homing of microglia to plaques and the phagocytosis uptake of Aβ. The precise mechanisms mediating the functions of sTREM2 require further investigation. Although our previous work suggests that sTREM2 may modulate microglial functions by binding to the cell surface receptor(s) [15], the identity of sTREM2 receptor(s) remains to be characterized. Since the sTREM2 fragment 41–81 is a more potent inducer of microglial activation than the full-length sTREM2, it is plausible that this truncated mutant has higher affinity to the potential sTREM2 receptor(s). Furthermore, the extracellular domain of TREM2 has been shown to bind a wide array of ligands, the sTREM2 fragment 41–81 being much shorter in length might have more specific interaction partners than the physiological form of sTREM2. Hence, using the sTREM2 fragment 41–81 for screening of sTREM2 receptor(s) might be superior to the full-length sTREM2 in terms of assay sensitivity and specificity.

Accumulating evidence suggests a protective role of sTREM2 against amyloid pathology and related toxicity. Higher CSF sTREM2 levels were correlated with increased gray matter volume in MCI patients [28]. Furthermore, an association between higher CSF sTREM2 levels and slower cognitive and clinical decline was observed in individuals with MCI or AD dementia [11]. More recently, it was shown that higher CSF levels of sTREM2 are associated with slower rates of Aβ accumulation as assessed by PET imaging on human AD brain and transgenic mouse models [13]. Interestingly, higher CSF sTREM2 was also associated with lower tau PET accumulation in AD patients, suggesting the possibility that sTREM2 plays an important role in Aβ-related tau pathology. Overall, these observations support a role of sTREM2 that protects against the development of key AD pathology. In our study, a sTREM2 fragment composed of 41-amino acids was more potent than the full-length sTREM2 protein in modulating microglial responses and mitigating the amyloid pathology. Hence, this minimal active sTREM2 fragment represents a promising candidate for AD therapy. In Parkinson's disease (PD) patients with abnormal CSF tau concentration, the CSF levels of sTREM2 are also positively associated with cognitive performance [29]. Although the roles of sTREM2 in PD-associated pathology remain to be determined, it is tempting to propose that increasing sTREM2 might be beneficial for PD. Taken together, the minimal active sTREM2 peptide defined in our current study might be applied for the treatment of multiple neurodegenerative diseases.

## Conclusions

In summary, we define a 41-amino acid sequence of sTREM2 that is sufficient for modulating microglial functions and ameliorating the amyloid-associated pathology. Importantly, this minimal sTREM2 sequence was more potent than the full-length sTREM2 in lessening the plaque load and the plaque-associated neurotoxicity. Our findings also suggest that the interaction of sTREM2 with Aβ is essential for enhancing microglial recruitment to the vicinity of an amyloid plaque and reducing the plaque load. Taken together, our data provide more insights into the mechanisms underlying sTREM2 function and the minimal active sTREM2 sequence represents a promising candidate for AD therapy.

## Data Availability

The datasets used in the current study are available from the corresponding author on reasonable request.

## Abbreviations

AD: Alzheimer’s disease
CSF: Cerebrospinal fluid
TREM2: Triggering receptor expressed on myeloid cells 2
sTREM2: Soluble TREM2
MCI: Mild cognitive impairment
PFA: Paraformaldehyde
CDR1: Complementarity-determining region 1
TUNEL: Terminal deoxynucleotidyl transferase-mediated dUTP nick end-labeling
HRP: Horseradish peroxidase
oAβ_1–42_: Oligomeric amyloid-β 1–42
Thioflavin-S: Thio-S
PD: Parkinson's disease
